# Cell type discrimination based on image features of molecular component distribution

**DOI:** 10.1038/s41598-018-30276-1

**Published:** 2018-08-06

**Authors:** Arno Germond, Taro Ichimura, Liang-da Chiu, Katsumasa Fujita, Tomonobu M. Watanabe, Hideaki Fujita

**Affiliations:** 1 Laboratory for Comprehensive Bioimaging, RIKEN Quantitative Biology Center, 6-2-3 Furuedai, Suita, Osaka 565-0874 Japan; 2 Department of Chemistry, the University of Tokyo, 7-3-1 Hongo, Bunkyo-ku, Tokyo 113-0033 Japan; 3 Department of Applied Physics, Osaka University, 2-1 Yamadaoka, Suita, Osaka 565-0871 Japan; 4 Waseda Bioscience Research Institute in Singapore (WABIOS), 11 Biopolis Way, #05-02 Helios, Singapore, 138667 Singapore

## Abstract

Machine learning-based cell classifiers use cell images to automate cell-type discrimination, which is increasingly becoming beneficial in biological studies and biomedical applications. Brightfield or fluorescence images are generally employed as the classifier input variables. We propose to use Raman spectral images and a method to extract features from these spatial patterns and explore the value of this information for cell discrimination. Raman images provide information regarding distribution of chemical compounds of the considered biological entity. Since each spectral wavelength can be used to reconstruct the distribution of a given compound, spectral images provide multiple channels of information, each representing a different pattern, in contrast to brightfield and fluorescence images. Using a dataset of single living cells, we demonstrate that the spatial information can be ranked by a Fisher discriminant score, and that the top-ranked features can accurately classify cell types. This method is compared with the conventional Raman spectral analysis. We also propose to combine the information from whole spectral analyses and selected spatial features and show that this yields higher classification accuracy. This method provides the basis for a novel and systematic analysis of cell-type investigation using Raman spectral imaging, which may benefit several studies and biomedical applications.

## Introduction

Fundamental research and applications in biological and biomedical fields increasingly rely on automated laboratory systems to perform cytological profiling. Automated systems provide the opportunity for systematic, accurate, and cost-reduced procedures for disease diagnosis, profiling of drug responses, and the production of cell lines such as stem cells^1,2^. Computer-assisted cytological profiling relies on extracting morphological features from cell images for use in classification^3,4^. Previous studies have primarily used brightfield (transmission) images or fluorescence images of subcellular structures to extract mathematical features^5,6^. Regardless of the chosen algorithms, high accuracy (70% or more) in discriminating cells or cell structures has been reported^5–7^.

**Figure 1:**
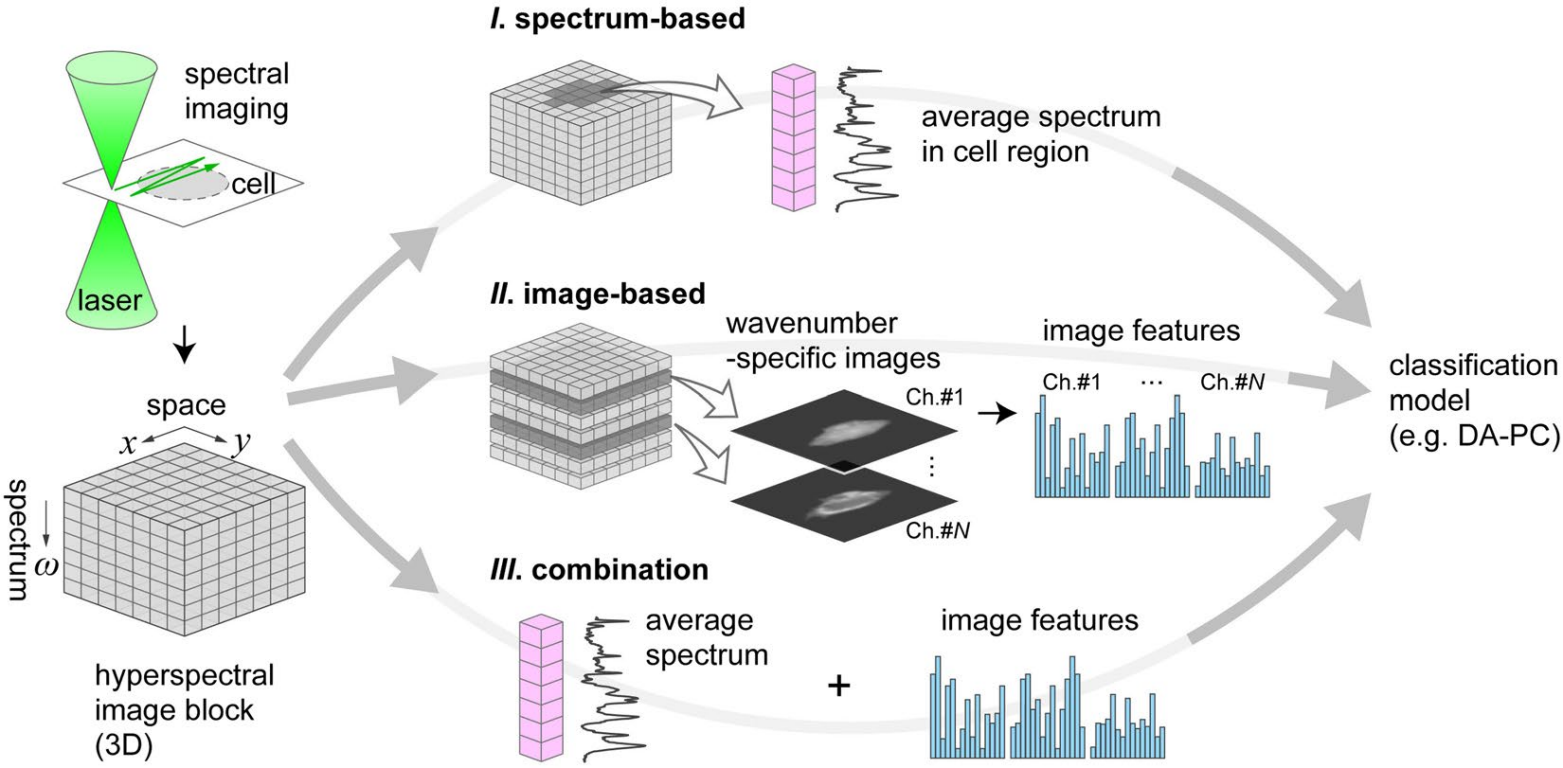
Overview of three different approaches to exploit information from Raman hyperspectral images prior to classification. (**I**) Spectrum-based approach. Cell information can be retrieved by calculating the average spectrum in the cell region. (**II**) Image-based approach. Various wavenumbers can be used to map the distribution of molecular compounds. From this set of images, image features can be computed using various algorithms (e.g., image transformation) to obtain a spatial frequency spectrum. The most relevant features can be used for classification. (**III**) Combined use of both the aforementioned methods prior to classification.

In contrast to the brightfield and fluorescence, vibrational spectroscopy gives image-contrast and information on many chemical structures and the composition of targeted samples in a single-exposure. In particular, Raman spectral imaging can achieve single-cell resolution without the need of labelling agents, which is especially relevant for the study of living cells, and medical and therapeutic applications^8^. A Raman hyperspectral image of an individual cell can be obtained by scanning the sample with a focused laser beam. The hyperspectral image is a x-y two-dimensional map where each pixel is associated with a spectrum of approximately one thousand wavenumbers (Fig. 1). A spectrum reflects various biomolecular compounds (e.g., lipids, protein, DNA, cytochrome c, nucleic acids, etc.). The average spectrum of a cell can be obtained by determining the cell’s area (Fig. 1I). In previous studies, our group and other groups demonstrated that the average spectrum from single cells do provide a reliable chemical fingerprint for the cells, and that variations in the peaks intensities of this spectrum allow to identify and classify the cell-types or cell-states in a reproducible manner^9–11^.

A hyperspectral image also allows one to reconstruct an image of a given molecular compound (i.e., wavenumber, or spectral band), thus giving a spatial pattern of its distribution within the cell (Fig. 1II). Previous Raman bioimaging studies reported that the distribution of specific molecular compounds could be used to quantify intracellular biological events of interest, such as the cytochrome activity^12^. Therefore, we envision that introducing Raman spectral images to extract explanatory variables for machine-learning would be a promising approach to achieve effective cell-state classification.

In this paper, we propose a novel, comprehensive method to classify living cells based on the mathematical patterns extracted from Raman hyperspectral images of single-cells (Fig. 1). In our method, we applied eleven image-transforms to Raman images in order to extract the mathematical features of the images. The image features are ranked by their Fisher scores according to their statistical importance. These features can be input into any kind of classifier for the purpose of discrimination. Using a dataset of hyperspectral images from three mouse cells lines, we demonstrate that the accuracy and robustness of the classification can increase when using an image pattern rather than an average spectrum representing the cell. Then, we demonstrate that the combination of both methods is also possible. The current study provides supporting evidence that our methodology can benefit the analysis of hyperspectral images in biological and biomedical studies.

## Materials and Methods

### Cell culture

Hepa1–6 and neuro2A were obtained from the RIKEN BioResource Center (BRC) cell bank, mouse mesenchymal stem cells (MSC) were purchased from Takara Bio, and Hepa1–6 and MSC were cultured in Dulbecco’s Modified Eagle’s medium (DMEM: 4.5 g/L glucose; Sigma-Aldrich, St. Louis, MO) supplemented with 10% Fetal bovine serum (FBS; Gibco) and antibiotics (1% penicillin-streptomycin; Sigma-Aldrich). Neuro2A was cultured in Minimum Essential Medium (MEM; Sigma-Aldrich) supplemented with 1% nonessential amino acids (Sigma-Aldrich), 10% FBS and antibiotics. To avoid a strong background scattering signal during Raman measurements, cells were seeded on a silica coverslip (SPI supplies, West Chester, PA) coated with 0.1% gelatin and cultured for three days.

### Raman spectral imaging and spectral pre-processing

Immediately before observation, the medium of cell cultures was replaced with warm PBS. All data were recorded with a custom-built slit-scanning Raman microscope based on an inverted microscope with an excitation wavelength of 532 nm for all observations. This setup was described previously^13^. A water-immersion objective lens with a 1.27 numerical aperture (NA) (40×, CFI Plan Apo IR, NIKON, Japan) was used as the objective lens. The Perfect Focus System remained active during all measurements. We used a line illumination system to perform the scanning of cells. The laser is shaped as a line which allow to obtain 400 spectra in a single exposure, considerably reducing the time for scanning living cells. The spatial resolution of one line is about 300 nm. The line-shaped focused laser beam was scanned over a 40 μm range with 120 lines. The exposure time for each line was 5 s with a laser intensity of 2.4 mW/μm^2^. Hyperspectral images were processed through cosmic-ray removal, background subtraction, baseline correction and normalization in the same way as done in our previous study^9^. Specifically, to determine which pixel compose a given cell, first we identified cells using the spectral information of protein peaks (1650cm^−1^) and lipid peaks (2850 cm^−1^) using threshold values. When image contrast was not sufficient, pixels were added manually. For each cell lines, 24 images were obtained.

### Construction of wavenumber-specific images and extraction of image features

A hyperspectral image of Raman scattering is composed of Raman spectra (1024 wavenumber points) in two-dimensions (Fig. 1, left). For each position, the intensities of a few selected Raman peaks (750 cm^−1^ for cytochrome, 1687 cm^−1^ for protein, and 2850 cm^−1^ for lipid) were fit to a Gaussian function. These three peaks, which represent three major biological compounds of cells, were selected to reconstruct grayscale images because they provided the best contrast of images. For cell segmentation, individual cell areas were manually delimited based on the Raman images at the protein peak (1687 cm^−1^), which exhibit relatively homogeneous distribution within a cell and reflect the cell shape accordingly. Computation of the image features was performed using an open-source software for image analysis called wndchrm, which was developed by Orlov and colleagues^14,15^. The algorithm extracts a generic set of numerical image content descriptors, including textures, statistical distribution of pixel values, and factors from polynomial decomposition of the raw image, as well as several image transforms of the raw image as previously described^14^. For each image constructed from the above peak intensities, 1025 features were obtained. We consider that the obtained ensemble of features is a signature of the image for the selected chemical compound present in the area of a segmented cell. For each cell, the image features from the three channels are then concatenated, yielding a single array of 3075 variables. All the variables (features) are standardized to have an average of 0 and standard deviation of 1. Each feature is assigned a weight corresponding to its informativeness^14^. We adopted the Fisher discriminant score as the feature weight, which can be conceptualized as the ratio of inter-group variance to the intra-group variances. The weighting method allows one to select the most informative features for classification, and a previous study discarded the features considered as less informative^15^. In this paper, we explored which number of features is required to achieve the best classification by comparing the performance of the model. Performance was evaluated using R^2^ entropy scores and −2 logLikelihood scores.

### DA-PC classification models

Our dataset contained 72 images of single living cells. As a test dataset, cells measured on a different day (independent experiment) were considered (hold-back proportion of 25%, so that 6 cells were used as test data for each cell line). Classification models were built with principal component analysis (PCA)^16^ followed by a discriminant analysis (DA), an approach we name here DA-PC. The DA-PC approach requires *a priori* knowledge of the observation groups (label) to train the model. Calculations were performed using the JMP software (JMP^®^, Version v11, SAS Institute Inc., Cary, US). The PCA allowed the spectra to be decomposed into a linear combination of loading vectors that quantitatively express the type or state of cells in a reduced number of dimensions. Principal components (PCs) were then selected and used as the DA input. Only PCs with a statistically significant approximate *F*-value (*p* < 0.001) were included in the subsequent DA. The approximate *F*-values were calculated following the Wilk’s Lambda and Hotelling-Lawley Trace as defined in the JMP suite. The DA assumes that each group has a multivariate normal distribution, and calculates the Mahalanobis distance, which is the distance of an observation from the mean of a group divided by the standard deviation along the direction vector. Quadratic DA was applied to consider that the intra-group covariance matrices are not assumed to be equal. For a given observation, the membership probabilities in each group are calculated based on the Mahalanobis distances, and the observation is classified to the group with the largest probability of membership. To evaluate the performance of all the DA-PC models, scores of the R^2^ entropy and negative log-likelihood (−2 logLikelihood) were considered. Briefly, the R^2^ entropy score, hereafter called R^2^, is a measure of fit that estimates how the fitted model performs better than the null model. The likelihood function, denoted L(β), is the product of the probability density functions (or probability mass functions for discrete distributions) evaluated at the observed data values. We use here the minimization of the negative log-likelihood (−2 logLikelihood) score. Detailed equations are described in the JMP suite. Better models should give higher R^2^ and lower −2 logLikelihood (Supplementary Table 1).

### Other machine-learning classification models

The performance of 3 well-known classifiers to discriminate cell lines from the features obtained from Raman images was compared. Specifically, we applied a Projection on Latent Structure (PLS-DA), a K-means predictive model and a Support Vector Machine (SVM). Like in the above DA-PCA approach, 6 cells were used as test data for each cell line (25% test data). Standardized images features were used as input. For cross-validation of the model, a Venetian blind cross-validation with 10 splits was applied. For PLS-DA, a model including 2 components was chosen. For K-means, 3 clusters were defined. For SVM, the model optimization is automatic. These analyses were performed using the Eigenvector software (Eigenvector Research Inc., Wenatchee, USA). While these models provide valuable alternatives, we choose to focus on the DA-PC model in this manuscript to perform the combination of Raman spectral data and image features as described below.

### Data availability

Data are made available on request.

## Results and Discussion

In the present study, we propose a new method to classify cells based on the features extracted from the spectral images of molecular components in single living cells. Below, we show that this method can be used as an alternative to the more conventional approach using the average spectrum. Then, we demonstrate that using image features can also complement the conventional approach, with the possibility to significantly improve the classification accuracy. An overview of our concept is summarized in Fig. 1.

### Spectral-based classification of three mouse cell lines

**Figure 2:**
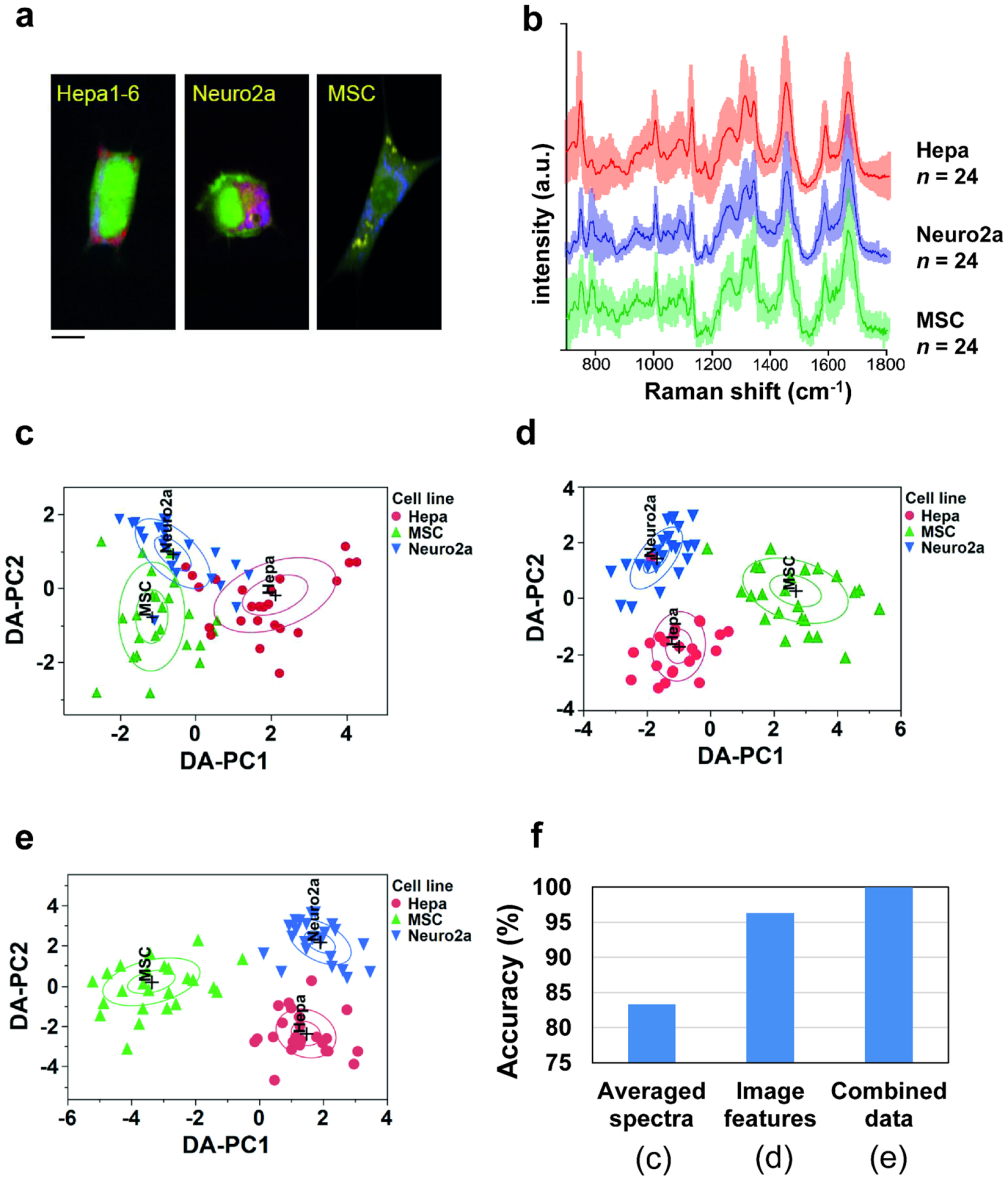
Comparison of three analytical approaches to Raman hyperspectral images of mouse cell lines. (**a**) Typical Raman images of three types of mouse cells (Hepa1–6, Neuro2a, and MSC) used in this study. Raman peaks at 750 cm^−1^ (cytochrome C), 1687 cm^−1^ (proteins), and 2850 cm^−1^ (lipids) are mapped in blue, green, and red, respectively. (**b**) Averaged Raman spectra of the three types of cells. Solid lines are the averaged spectra and the faded ribbons show the standard deviation for each spectrum. The average and SD were obtained from 24 cells for each cell-type. (c-e) Discrimination of the cell types using the DA-PC models. DA-PC score plots on the first two DA-PC dimensions obtained with approach I (**c**), II (**d**), and III (**e**). The inner ring and outer ring represent 95% and 50% confidence intervals, respectively. (**f**) Comparison of the classification accuracies achieved by the three discrimination models shown in (**c**–**e**).

Three types of mouse cell lines were purposely chosen for their biological importance and their different morphologies. Hepa1–6 (Hepa) is a cell line derived from mouse hepatoma, neuro2a (N2a) is a neuroblastoma cell line, and mesenchymal stem cells (MSC) are primary cells. Figure 2a shows spectral images of these cells as reconstructed from their spectral features and coloured according to three different wavenumbers (i.e., channels), namely cytochrome (750 cm^−1^), proteins (1687 cm^−1^), and lipids (2850 cm^−1^).

One conventional approach to analyse Raman spectral images of cells is to delimit the area of a given cell, then calculate an average spectrum across the pixels contained in this area to obtain a global chemical signature (Fig. 2a). Because spectral averaging reduces the variation within a cell, it also reduces the complexity of the analysis, or can benefit cases for which the image has a limited spatial resolution or pixel resolution. In many studies, the average spectrum of the whole cell, or eventually of the nucleus and cytoplasm, has been shown to be informative to classify cells by types or states^9–11^. Likewise, we apply this approach to verify the classification accuracy when using the averaged spectra from single cells. After delimiting each cell and performing appropriate background subtraction and pre-processing, the averaged Raman spectra (878 variables) were used as the input of our DA-PC model. In this particular dataset, it is interesting to note that the cells, despite their different morphologies, have rather similar averaged spectra (Fig. 2b). The DA-PC model shown in Fig. 2c exhibited an accuracy of 83.3%, yielding an R^2^ score of 0.610 and a −2 logLikelihood score of 45.361. (Supplementary Table 1). We found these scores were the worst when compared to all other models evaluated in this study (Supplementary Table 1). This initial result suggests that this approach does allow cell type classification, but it may be limited by similarities in the molecular profiles averaged across different cells.

### Image-based classification of spectral image of cells

**Figure 3:**
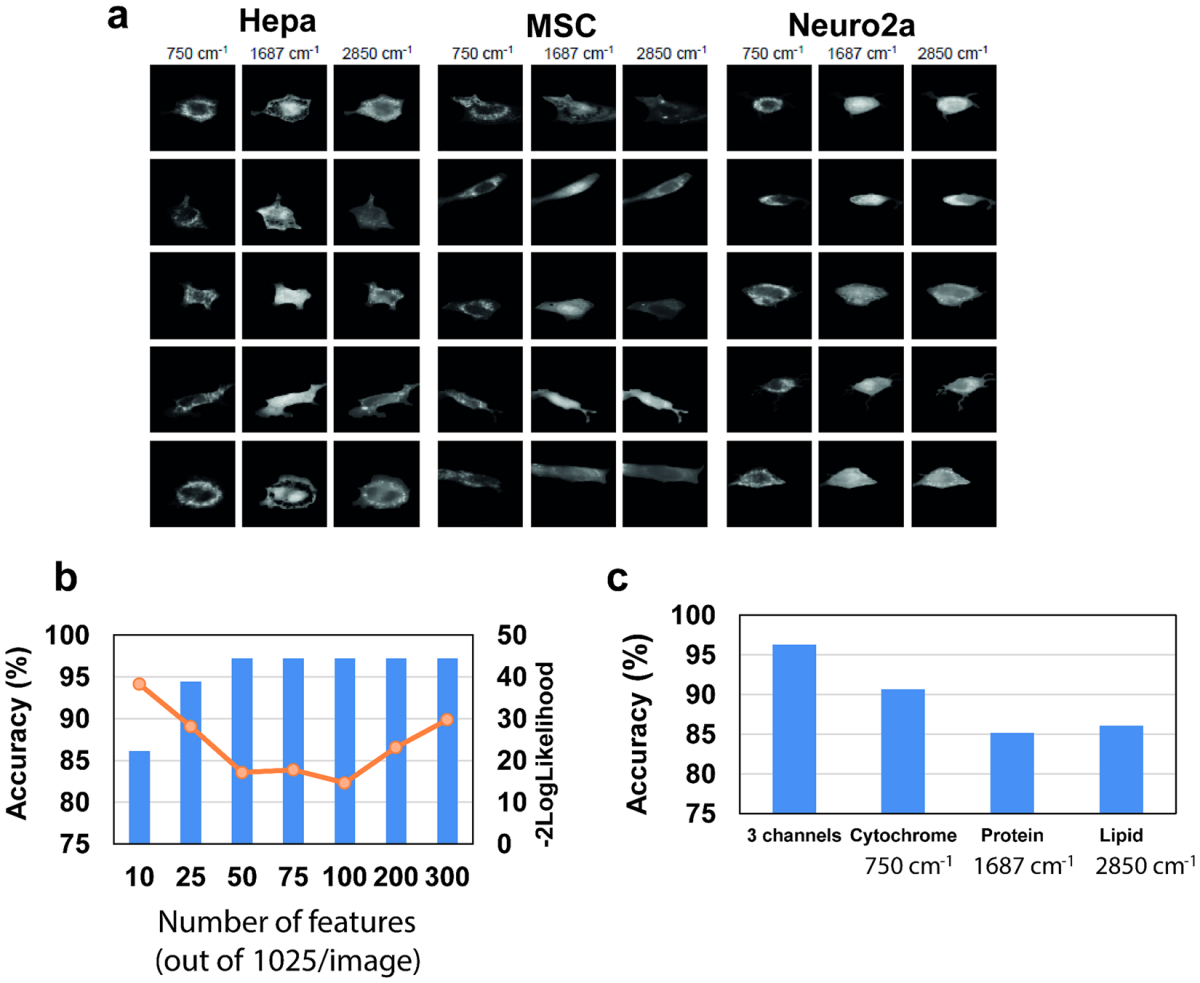
Feature extraction from spectral images at specific wavenumbers, and comparison of image-based classification results. (**a**) Example images showing the distribution of cellular compounds for three cell lines at three different channels associated to cytochrome (750 cm^−1^), proteins (1687 cm^−1^), and lipids (2850 cm^−1^). (**b**) Comparison of classification models when using the top 10 to top 300 features concatenated from the three channels. The overlaid curve shows the −2 logLikelihood score. The model with 100 features showed the highest accuracy and lowest −2 logLikelihood score. (**c**) Comparison of classification models computed using the top 100 features for three separate or concatenated channels. The best model was obtained when the three channels were combined together, which model includes 100 features.

Here, we choose 3 different channels (i.e., spectral bands), each representing a different molecular bond associated to cytochrome (750 cm^−1^), proteins (1687 cm^−1^), and lipids (2850 cm^−1^) to reconstruct images that are specific to these compounds. These molecular compounds have a strong biological importance and the local maximum around these spectral wavenumbers also allow to obtain the best contrast on images. Examples of a few image cells are shown in Fig. 3. These images are then used to calculate the mathematical features of spatial patterns using eleven mathematical algorithms described previously by Orlov and colleagues^14^. We used the Fisher scores as a measure to evaluate the contribution of each feature to cell type classification. Therefore, the features with the highest Fisher score, which are likely to best explain the variation of patterns across the samples, can be selected as an input of any classification model, such as the DA-PC model used here.

It is likely that the optimum number of image features required to achieve the best classification is specific to each dataset, and therefore the adequate number of features must be carefully assessed beforehand. In their study, Shamir and colleagues suggested to remove 35% of the features that are associated with the lowest Fisher scores, as these are considered to be less informative^15^. Here, we explored the number of features required to achieve accurate classification, as evaluated by the R^2^ and −2 logLikelihood scores described above. Classification models were built using 10, 25, 50, 75, 100, 200 and 300 features associated with the highest Fisher scores (Fig. 3b, Supplementary Table 1). Our results showed that the use of the top 50, 75 or 100 features was sufficient to achieve high classification accuracy for this dataset (97.5%) (Fig. 3b, Supplementary Table 1). However, the highest R^2^ score and the lowest −2 logLikelihood score were obtained when using the top 100 features (Supplementary Table 1), suggesting that this number of features provided a more robust model. Therefore, we choose to keep the 100 top features in subsequent analyses of this dataset. The list of these features and their associated Fisher scores are shown in Supplementary Fig. S1.

Using the top 100 features out of 3075 mathematical features calculated for each cell (across the three channels), the three mouse cell lines were classified using the DA-PC model (Fig. 2d). The DA-PC model exhibited an accuracy of 96.3%, for an R^2^ of 0.87 and a −2 logLikelihood of 15.453. Only two cells were misclassified (1 Hepa and 1 MSC cell) and were identified as belonging the group of the neuro2a cells. This classification results being based on the mathematical features of pattern distribution, it suggests that these two cells were mistaken as neuro2a cells due to similarities in the pattern distribution of molecular compounds at the chosen three channels. It worth noticing that the image-based model performed better than when using an average over the spectral data (Fig. 2c). Thus, the image-feature extraction method gave us the opportunity to classify the cells from a reduced number of channels with a high accuracy.

### One channel versus multiple channels

In most cases, the user has no *a priori* knowledge about which spectral wavenumber should be more relevant to describe the biological system under study. Here, we compared how the information extracted from each channel defined above (cytochrome, lipids, and proteins) could perform in discriminating the three mouse cell lines. For each channel, 100 features with highest Fisher scores were extracted and used to build the DA-PC models. The results are presented in the Fig. 3c. While the accuracy for each independent single channel (namely, cytochrome, protein, or lipid) was overall greater than or equal to 85%, the best single-channel model was obtained for the cytochrome-related wavenumber. The model constructed with individual channels however performed less well than the model with the combined information from three channels (Fig. 3c). The combined model includes 100 features, which was also compared to models with other number of features (Fig. 3b).

Cytochrome activity is particularly relevant in cell biology and it is interesting to see that the pattern of distribution of this molecular compound resulted in a better classification. Changes in the cytochrome c distribution can be associated to the release of cytochrome c from mitochondria, an indicator of apoptosis^12^, or to an increase of cytochrome proteins, which play a role in glycolytic activity and cell proliferation^17^. Hence, the differences in cytochrome distribution observed across the observed cell lines might account for differences in their metabolic states link to glycolytic activity or proliferation. Unfortunately, at the time of the experiment, we did not verify this hypothesis by conventional methods. In a more general perspective, by comparing the classification results obtained from various independent wavenumbers, one can identify the wavenumber the most informative for classification, which may help to describe their biological system or generate testable hypotheses.

Alternatively, when *a priori* knowledge on the system is available, the spectral channels that are thought to be biologically-relevant can be selected to reconstruct the images prior to classification. The rich information of label-free Raman spectroscopy gives this advantage to select, after the measurement is done, the wavenumbers that are known to be associated with a specific cell type or cell state. As a case in point, a previous study pointed out the importance of the protein related peak at 757 cm^−1^ and the nucleic-acid related peak at 784 cm^−1^, which ratio has been used in discriminating and predicting the differentiated state of embryonic stem cells^18,19^. Many other wavenumbers can be envisaged. For example, previous studies reported that the possibility to monitor the degree of unsaturation and transition temperatures of constituent lipids in microalgae^20^, or cell cycle and cell confluency in human cancer cells^21^. In such cases for which wavenumber of interest are known, the classification could be tightly correlated to the biological state of the cells, thus becoming more relevant from a biological perspective.

### Applicability of other classifiers for cell image analysis

To our best knowledge, very few studies have been taking advantage of machine learning classifiers to extract features from Raman images and perform the automated discrimination of cells. It is likely that depending on the dataset, one kind of classifier may perform better than others. Although it is not the objective of this paper to do an extensive investigation of how different models perform, we provide in the Supplementary Table 2 a comparison the classification results of three well-known classifiers as an example. Specifically, we applied a Projection on Latent Structure (PLS-DA), a K-means predictive model and a Support Vector Machine (SVM) models to identify the three cell lines based on the analysis of top 100 image features across three channels. Results are described in Supplementary Table 2.The DA-PCA model shown in Fig. 2d had misclassified two cells among tested data. Unfortunately, the software we used for these models did not provided the same indicators as we obtained for the DA-PCA models. However, by comparison to DA-PCA, we found that these three other kinds of model performed generally very well in classifying cell types as judged by the low error rate on the R2 for predicted (test) dataset. The SVM model had the best results during the training data, and the K means model the best result on the test dataset. These results must however be taken cautiously due to the small size of our dataset. The point we want to underline is, however, that other classifiers can be used to perform the classification of living cells using Raman spectral images, and that they can perform as well if not better than DA-PCA.

### Combining spectral and spatial information

In some cases, there is the possibility that the image information is not informative enough to discriminate the cell types or cell states. To overcome this issue, we envision that the combination of both spectral and image information could be useful to increase the accuracy of classification, because they provide complementary aspects of the cell phenotypes. Here, we choose to apply the DA-PCA approach to combine both types of information to test this hypothesis. The advantage of the DA-PCA approach by comparison to the other classifiers mentioned above (such as PLS-DA), is that it allows to combine data of different nature through the PC components. Here, standardized PC components calculated independently from spectral and image information were included in the model. Then, a stepwise selection of the PCs components was performed based on their discriminant scores and *p* values (see Materials and Methods). In this model, 3 components from the spectral dataset and three components from the image features calculated from image transformation were selected, suggesting that these two modes of information provide unique aspects of the cells. The resulting DA-PC plot of Fig. 2e shows that the model accurately discriminated the three cell lines, with an accuracy of 100%, an R^2^ value of 0.99 and a −2 logLikelihood of 1.096. The scores show that this model performed better compared to the DA-PC models of Fig. 1 (Supplementary Table 1). In Fig. 2d, we note that the Hepa cell and the MSC cell were placed close to the Neuro2a group in two dimensional spaces, showing that image information alone was not sufficient to discriminate these two cells from neuronal cells. However, in the DA-PC plot of Fig. 2e, these cells were closer to their original group, demonstrating how the contribution of the spectral information complemented the image information to identify these cells back to their original group. This result showed that, for our dataset, the combination of (i) averaged spectral information and (ii) the spatial information extracted from the distribution patterns of the molecular compounds may lead to a more robust classification. However, we acknowledge it may be not the case for other datasets.

### Comparison to other imaging methods

Previous studies have focused on the use of brightfield or fluorescent-labelled images of cells prior to classification^6^. Fluorescent studies can be limited by the number of resolvable colours (up to nine using complex instrumentation)^22^, although only a few channels can be used simultaneously to monitor biological phenomenon of interests. However, when comparing a very large number of cell lines and/or cellular states, the number of resolvable colour can arguably become a limiting factor for the purpose of cell discrimination. In this regard, label-free spectroscopy can provide as much channels as needed to achieve a good classification, although further investigations using large datasets must be conducted to verify this assumption. Moreover, label-free Raman spectroscopy can circumvent the limitations of impermeability, toxicity, and specificity of the fluorescent reporters. Other technical advantages have been mentioned in previous studies. For example, in the study case of lipidomics, Raman spectroscopy allows to circumvent the need for lipid extraction and analysis, which is slow and invasive^20^. It must be stressed, however, that the above imaging techniques all provide different information with different temporal resolution and sensitivity, therefore they are all valuable regarding their respective applications.

## Conclusion

In this study, we demonstrated that the presented approaches are particularly efficient to perform and improve the classification of single living cells (Fig. 2). Although this study is limited to a single dataset of limited sample size, our approach is systematic and can be adapted to other study types of classifiers and datasets. The statistical analysis of cell images allows to quantify how molecular compounds distributes within single cells, which may help in return to generate hypotheses about why the considered cell-lines or cell states would differ for this given compound. Alternatively, depending on the application, we described the possibility to choose spectral wavenumbers that are known to specifically relate with the biology of the studied system. This may be relevant for a number of biomedical or industrial application. Future developments may improve even more the quantity of information that can be extracted from hyperspectral information. In a future work, we would like to combine the information from brightfield, contrast-phase, or fluorescence images with Raman spectral information and see how each method or their combination can contribute to the discrimination of cells. Overall, the present study provides a basis for further investigations in exploiting the full potential of information from spectral images.

## Electronic supplementary material

Supplementary Information.
