# Supplementary material for: Cell type discrimination based on image features of molecular component distribution
Source: Sci Rep. 2018 Aug 6;8:11726. doi: 10.1038/s41598-018-30276-1 (PMC6079059; doi:10.1038/s41598-018-30276-1)
Supplement: Supplementary file 1 — Supplementary Information. [file 41598_2018_30276_MOESM1_ESM.docx]

**Cell type discrimination based on image features of molecular component distribution**

Arno Germond^1^, Taro Ichimura^1^, Liang-da Chiu^2^, Katsumasa Fujita^3^, Tomonobu M. Watanabe^1*^, Hideaki Fujita^1,4^*

^1^*Laboratory for Comprehensive Bioimaging, RIKEN Quantitative Biology Center, 6-2-3 Furuedai, Suita, Osaka 565-0874, Japan*

^2^*Department of Chemistry, the University of Tokyo, 7-3-1 Hongo, Bunkyo-ku, Tokyo 113-0033, Japan*

^3^*Department of Applied Physics, Osaka University, 2-1 Yamadaoka, Suita, Osaka 565-0871, Japan*

^4^*Waseda Bioscience Research Institute in Singapore (WABIOS), 11 Biopolis Way, #05-02 Helios, Singapore 138667, Singapore*

**Supplementary information**

**Supplementary Tables**

| **Figure** | **Models** | **Var.** | **Accuracy** | **% error** | **R^2^** | **-2log Likelihood** | **Wilk's Lambda Test Value** | **Wilk's Lambda *p* Value** | **Hotelling-Lawley Test Value** | **Hotelling-Lawley *p* Value** |
| --- | --- | --- | --- | --- | --- | --- | --- | --- | --- | --- |
| Fig. 2c | Spectra | 868 | 83.3 | 16.7 | 0.618 | 45.361 | 0.233 | 0.001 | 2.362 | 0.001 |
| Fig. 3b | Image RGB | 10 | 88.9 | 11.1 | 0.743 | 30.468 | 0.144 | 0.001 | 3.473 | 0.001 |
| Fig. 3b | Image RGB | 25 | 94.4 | 5.6 | 0.809 | 22.698 | 0.121 | 0.001 | 3.788 | 0.001 |
| Fig. 3b | Image RGB | 50 | 96.3 | 3.7 | 0.861 | 16.527 | 0.081 | 0.001 | 5.047 | 0.001 |
| Fig. 3b | Image RGB | 75 | 96.3 | 3.7 | 0.866 | 16.179 | 0.072 | 0.001 | 5.587 | 0.001 |
| Fig. 2d,3b,3c | Image RGB | 100 | 96.3 | 3.7 | 0.870 | 15.433 | 0.073 | 0.001 | 5.712 | 0.001 |
| Fig. 3b | Image RGB | 200 | 96.3 | 3.7 | 0.869 | 15.601 | 0.052 | 0.001 | 7.115 | 0.001 |
| Fig. 3b | Image RGB | 300 | 94.4 | 5.6 | 0.811 | 22.485 | 0.080 | 0.001 | 5.382 | 0.001 |
| Fig. 3c | Image Cytochrome | 100 | 90.7 | 9.3 | 0.809 | 22.611 | 0.094 | 0.001 | 5.421 | 0.001 |
| Fig. 3c | Image Lipid | 100 | 86.1 | 13.9 | 0.740 | 40.556 | 0.186 | 0.001 | 3.497 | 0.001 |
| Fig. 3c | Image Protein | 100 | 85.2 | 14.8 | 0.716 | 33.666 | 0.187 | 0.001 | 2.790 | 0.001 |
| Fig. 2e | Spectra + Image RGB (100) | 968 | 100.0 | 0.0 | 0.991 | 1.096 | 0.031 | 0.001 | 9.682 | 0.001 |
|  |  |  |  |  |  |  |  |  |  |  |

**Supplementary Table 1. Comparison of the performances of the DA-PC models used in this study. Abbrevation Var. stands for the number of variable in each model.**

| **Model** | **Model complexity** | **Cell Type** | **Cal. Sensit.** | **CV Sensit.** | **Predict. Sensit.** | **Cal. Specif.** | **CV Specif.** | **Predict. Specif.** | **R2 Cal.** | **R2 CV** | **R2 Predict.** |
| --- | --- | --- | --- | --- | --- | --- | --- | --- | --- | --- | --- |
| SVM | number of | Hepa | 1 | 1 | 0.80 | 1 | 1 | 1 | 1 | 1 | 0.90 |
|  | SVs: 33 | MSC | 1 | 0.95 | 1 | 1 | 1 | 1 | 1 | 0.97 | 1 |
|  |  | Neuro2a | 1 | 1 | 0.80 | 1 | 0.97 | 0.90 | 1 | 0.99 | 0.95 |
|  |  |  |  |  |  |  |  |  |  |  |  |
| KNN | Cluster: 3 | Hepa | 1 | 1 | 1 | 1 | 0.95 | 1 | 1 | 0.89 | 1 |
|  |  | MSC | 1 | 0.79 | 1 | 1 | 0.97 | 1 | 1 | 0.83 | 1 |
|  |  | Neuro2a | 1 | 0.95 | 1 | 1 | 0.95 | 1 | 1 | 0.92 | 1 |
| PLS-DA | 2 LVs | Hepa | 1 | 1 | 0.80 | 1 | 1 | 0.70 | 1 | 1 | 0.75 |
|  |  | MSC | 0.95 | 0.90 | 1 | 0.95 | 0.92 | 1 | 0.95 | 0.91 | 1 |
|  |  | Neuro2a | 0.95 | 0.95 | 0.80 | 0.97 | 0.95 | 0.90 | 0.96 | 0.95 | 0.85 |

**Supplementary Table 2. Comparison of the three machine-learning classifiers other than DA-PCA for the discrimination of cell lines.** Single Vector Machine (SVM), predictive K-means clustering (K means), and Projection on Latent Structure Discriminant analysis (PLS-DA) were applied on the dataset of top 100 image features obtain from Raman hyperspectral images. The comparison describes in detail how perform the model during calibration, cross-validation and prediction using test data. For each strain, calibration/training data included 18 samples and test data included 6 samples. Sensitivity measures the proportion of actual positives that are correctly identified as such. Specificity measures the proportion of actual negatives that are correctly identified as such. DA-PCA could not be compared to theses model because the analyses were performed in different software/packages that do not provide the same indicators to allow model comparison. Abbrevations stand for: CV: cross validation, Cal.: calibration, Sensit. Sensitivity, Specif. specificity, Predict. Predicted test data.

**Supplementary Figures**


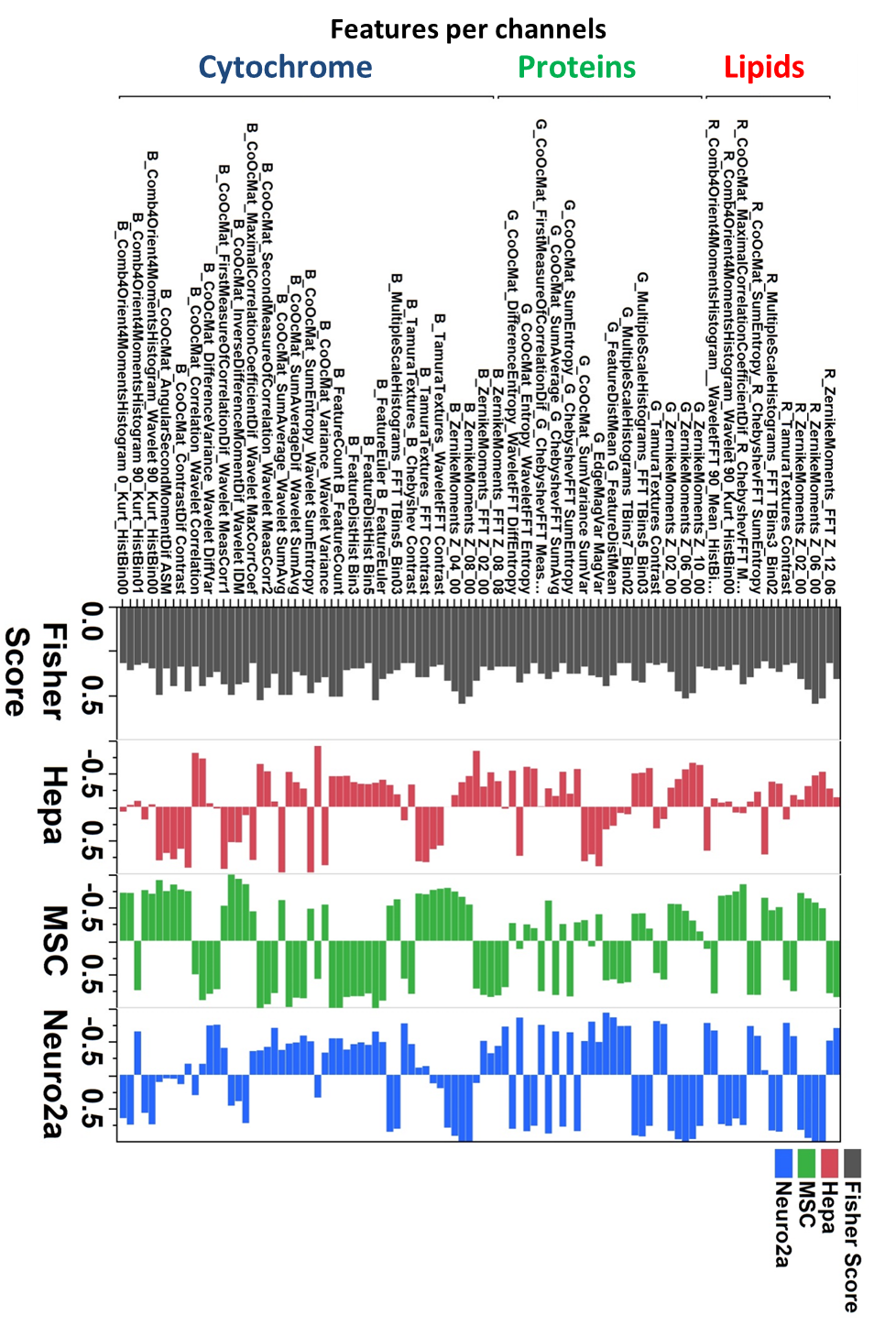


**Supplementary Figure 1. Features selection from hyperspectral images built from the combination of 3 channels representing the distribution of three molecular compounds of biological importance.** Features calculated from the image-transform (using eleven algorithms) for each channel were selected by their Fisher score, and only the features with the top 100 scores are shown. The figure shows the respective contribution of each channel (Cytochrome, Protein and Lipids) in selecting the features. The number of features associated to the cytochrome channel is dominant (approximately 50%). Moreover, we show how each feature respond differently for each cell lines, suggesting these features capture the differences in the spatial pattern of the distribution of molecular compounds. These differences are likely to contribute in the classification of the cell lines in classification models (such DA-PC), as shown in Fig. 2d.
